# Performance Evaluation of the BZ COVID-19 Neutralizing Antibody Test for the Culture-Free and Rapid Detection of SARS-CoV-2 Neutralizing Antibodies

**DOI:** 10.3390/diagnostics11122193

**Published:** 2021-11-25

**Authors:** Bo Kyeung Jung, Jung Yoon, Joon-Yong Bae, Jeonghun Kim, Man-Seong Park, Suk Yong Lee, Chae Seung Lim

**Affiliations:** 1 Department of Laboratory Medicine, Dankook University College of Medicine, Cheonan 31116, Korea; lovegodmother@hanmail.net; 2 Department of Laboratory Medicine, College of Medicine, Korea University, Seoul 08308, Korea; malarim@korea.ac.kr; 3 Department of Microbiology, Institute for Viral Diseases, College of Medicine, Korea University, Seoul 02841, Korea; harbe3103@korea.ac.kr (J.-Y.B.); totspurs@daum.net (J.K.); ms0392@korea.ac.kr (M.-S.P.); 4 Creativity Lab., Creativity & Innovation Center, Samsung Electronics, Suwon 16677, Korea; syren.lee@diavision.co.kr

**Keywords:** SARS-CoV-2, COVID-19, neutralizing antibodies, RDT, ELISA

## Abstract

Rapid and accurate measurement of SARS-CoV-2 neutralizing antibodies (nAbs) can aid in understanding the development of immunity against COVID-19. This study evaluated the diagnostic performance of a rapid SARS-CoV-2 nAb detection test called the BZ COVID-19 nAb test BZ-nAb (BZ-nAb; BioZentech). Using the 90% plaque-reduction neutralization test (PRNT-90) as a reference, 104 serum specimens collected from COVID-19-positive and -negative patients were grouped into 40 PRNT-90-positive and 64 PRNT-90-negative specimens. The performance of the BZ-nAb was compared with that of the cPass surrogate virus neutralization test (cPass sVNT; Genscript). The BZ-nAb showed a sensitivity ranging from 92.5%–95.0% and specificity ranging from 96.9%–100%, whereas cPass sVNT showed a sensitivity of 100% (95% confidence interval (CI) 90.5%–100%) and specificity of 98.4% (95% CI, 91.6%–100%). The dilution factor obtained with PRNT-90 showed a stronger correlation with the percent inhibition of cPass sVNT (r = 0.8660, *p* < 0.001) compared with the test and control line ratio (T/C ratio) of the BZ-nAb (r = −0.7089, *p* < 0.001). An almost perfect agreement was seen between the BZ-nAb and cPass sVNT results, with a strong negative correlation between the BZ-nAb T/C ratio and cPass sVNT percent inhibition (r = −0.8022, *p* < 0.001). In conclusion, the diagnostic performance of the BZ-nAb was comparable to that of the cPass sVNT, although the BZ-nAb had a slightly lower sensitivity.

## 1. Introduction

Coronavirus disease 19 (COVID-19), caused by severe acute respiratory syndrome coronavirus 2 (SARS-CoV-2), has been global pandemic disease since March of 2020. Immunity against SARS-CoV-2, induced either by infection or vaccination, has been shown to protect against subsequent reinfection and/or reduce the risk of developing a severe form of the disease [1,2]. Previous studies have shown that neutralizing antibodies (nAbs) against the receptor-binding domain (RBD) of the spike (S) glycoprotein of SARS-CoV-2 provide protective immunity against COVID-19 [3,4,5]. The nAb response against SARS-CoV-2 has been shown to be associated with patient survival, and the titer value is predictive of the extent of immunity developed by the host [6,7]. With the introduction of multiple SARS-CoV-2 vaccines, development of the nAb response and neutralization titers have been used to quantify the immunogenicity of vaccines in clinical trials [8,9,10].

nAb-testing assays conventionally include live-cell neutralization tests, such as the plaque reduction neutralization test (PRNT) and microneutralization assay. Traditionally, both PRNT and the microneutralization assay have been used to quantify the nAb response; however, the PRNT has been found to be more sensitive than the microneutralization assay for measuring SARS-CoV-2-specific nAbs [11,12]. These live-cell neutralization tests are limited by the need to be performed in a biosafety level (BSL) 3 laboratory and their small throughput, in addition to the high cost and skill required to perform them. A pseudovirus neutralization test using a pseudovirus expressing the SARS-CoV-2-specific S glycoprotein [13] can be performed in a BSL-2 facility; however, it requires the maintenance of virus cultures, which is not feasible in terms of the required facilities, cost, and resultant throughput. 

A blocking ELISA called the cPass surrogate virus neutralization test (sVNT) (GenScript, Piscataway, NJ, USA) is also commercially available. cPass sVNT is designed to specifically measure the levels of nAbs against the RBD of the S glycoprotein of SARS-CoV-2 and assesses the neutralizing capacity of nAbs, enabling the detection of functional SARS-CoV-2 neutralization activity [14]. The cPass sVNT results showed high agreement with those of live-cell neutralization detection methods [14,15,16]. In contrast to live-cell neutralization tests, cPass sVNT is performed without the need for virus cultures and requires only a few hours to complete. However, since cPass sVNT is an ELISA, specialized equipment for optical density measurements is still required.

Recently, rapid diagnostic tests (RDTs) for SARS-CoV-2 nAb detection, such as the BZ COVID-19 nAb test (BZ-nAb; BioZentech, Seoul, Korea), have been developed. The BZ-nAb is an immunochromatographic assay involving two components: the purified RBD of S protein and the angiotensin-converting enzyme 2 (ACE2) receptor. Similar to the cPass sVNT, the BZ-nAb also detects SARS-CoV-2 nAbs by detecting the degree of inhibition of the RBD-ACE2 interaction by nAbs in the sample. Theoretically, any immunoglobulin isotype targeting the RBD can be detected by the BZ-nAb. The BZ-nAb can be performed within 10–15 min, and the test results are obtained by the visual interpretation of the test line [17]. 

The purpose of this study was to evaluate the performance of the BZ-nAb to detect SARS-CoV-2 nAbs in serum samples of COVID-19-positive and -negative patients. The performance of the BZ-nAb was compared with that of cPass sVNT, which was used as the reference test for SARS-CoV-2 nAb detection. In addition to visual interpretation, the signal intensity of each line (both test and control) was analyzed using a smartphone-based image analysis application (SIA). With the development of mobile medical applications for automated interpretation of RDT results, analyzing RDTs with these applications has been reported to provide improved and reliable results [18,19,20]. A pilot study of SIA evaluation using different immunochromatographic assay and results showed acceptable performance (100% sensitivity and 97.8% specificity) when compared to visual interpretation results (data not shown). Our performance evaluation of the BZ-nAb involved the evaluation of two kinds of results, one obtained by visual interpretation, and the other measured by the SIA. The correlation between the results of the BZ-nAb and those of cPass sVNT was also determined. 

## 2. Materials and Methods

### 2.1. Sample Collection and Preparation

Serum samples from COVID-19-positive and -negative patients were collected from the Korea University Guro Hospital from March 2020 to December 2020. Patients were confirmed to be COVID-19-positive using real-time polymerase chain reaction (RT-PCR)-mediated detection of SARS-CoV-2. Patients with no reported history of COVID-19, were not vaccinated against SARS-CoV-2, and confirmed negative upon RT-PCR testing were designated COVID-19-negative patients. A total of 104 samples (45 COVID-19-positive samples and 59 COVID-19-negative samples) were collected from 100 patients (47 males and 53 females). Two samples with different intervals from the onset of symptoms were collected from four COVID-19 patients. The median age of patients was 63 years (ranged from 17 to 88 years). For COVID-19-positive patients, the median time after symptom onset was 17 days (ranged from 0 to 66 days). The presence of nAb against SARS-CoV-2 was confirmed using the PRNT, which was the reference method in this study, and serum samples were grouped according to these results. Serum samples were stored at −80 °C until analysis. Three samples out of a total of 104 samples were not subjected to cPass sVNT due to insufficient sample volume. This study was approved by the Institutional Review Board of the Korea University Guro Hospital (2021GR0146).

### 2.2. PRNT

The PRNT was performed as described previously [21]. Briefly, serum samples were diluted 1:10 using phosphate-buffered saline and serially diluted by two-fold to 1:160. A total of 100 µL of diluted serum was mixed with an equal volume of approximately 100 plaque-forming units of SARS-CoV-2 (BetaCoV/Korea/KCDC03/2020, NCCP 43326), resulting in final titers ranging from 1:20 to 1:320, and incubated for 1 h at 37 °C and 5% CO2. Vero cell plates (NEST Scientific, SPL Life Science, Pochen, Korea) were inoculated with the serum-virus mixtures, incubated for 1 h at 37 °C and 5% CO2 and overlaid with agar for 72 h at 37 °C and 5% CO2. Then, the plates were stained using 0.5% crystal violet, and the number of plaques was enumerated. A 90% plaque-reduction neutralization (PRNT-90) was the maximum dilution that resulted in a 90% reduction in plaques when compared with that of control plates. Samples with PRNT-90 titer ≥1:20 was considered PRNT-90-positive, and the remaining were considered PRNT-90-negative.

### 2.3. cPass sVNT

The cPass sVNT (GenScript, Piscataway, NJ, USA) was performed according to the manufacturer’s instructions. Serum samples were diluted 1:10 with the sample dilution buffer provided in the kit. The diluted samples were mixed with an equal volume of horseradish peroxidase-conjugated recombinant SARS-CoV-2 RBD fragment solution diluted with RBD dilution buffer. Next, 100 μL of this solution was added to the 96-well plate coated with the human ACE2 receptor and incubated for 30 min at 37 °C. The plate was washed four times with the provided wash solution. Then, 3,3′,5,5′-tetra-methylbenzidine was added to each well and incubated for 15 min in the dark, followed by addition of the stop solution. Optical density at 450 nm was measured and compared to that of the control wells. Percentage signal inhibition was calculated, and a cutoff ≥30% was considered positive, according to the manufacturer’s instructions.

### 2.4. BZ-nAb

**Figure 1:**
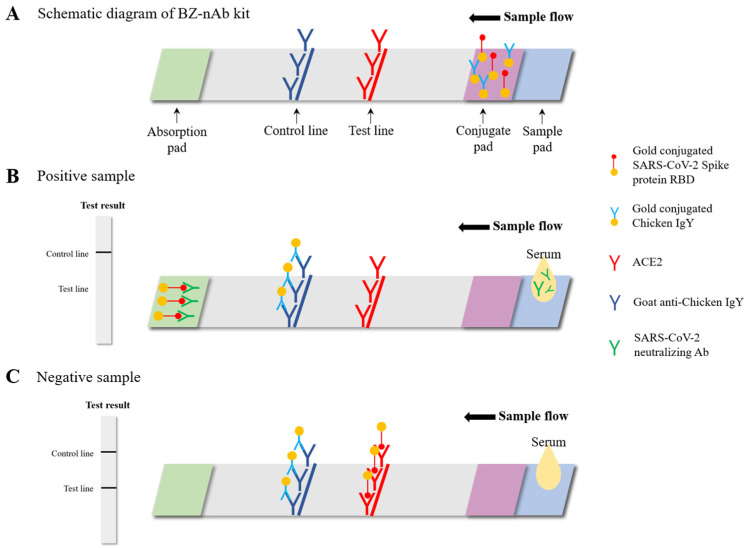
BZ-nAb test kit. (**A**) Schematic diagram of the BZ-nAb is shown. The diagram of SARS-CoV-2 neutralizing antibody positive (**B**) and negative (**C**) samples. In SARS-CoV-2 nAb positive samples, the RBD-ACE2 interaction in the test line are inhibited by nAbs in the sample, and vice versa in the negative sample.

The BZ-nAb is an immunochromatographic assay involving two components: the Gold conjugated SARS-CoV-2 S protein RBD and the ACE2 receptor. For internal control, Gold conjugated Chicken IgY and goat anti-chicken IgY are included (Figure 1A). The BZ-nAb detects SARS-CoV-2 nAbs by detecting the inhibition of the RBD-ACE2 interaction by nAbs in the sample (Figure 1B,C). 

A total of 40 μL of serum and 90 μL of RBD-containing buffer was added to the specimen well of the BZ-nAb cassette (BioZentech, Seoul, Korea). After 13–15 min of incubation, the intensities of the control and test lines were visually compared, and a sample was considered positive as follows: (1) absence of the test line with presence of the control line. (2) The intensity of test line lower than that of the control line (Figure S1). The line intensity is inversely proportional to the nAb titer. 

The signal intensities of BZ-nAb were also analyzed using an SIA (Figure S1). Immediately after the visual interpretation of BZ-nAb, images were captured using a smartphone camera (Galaxy S20 plus, Samsung), and the intensity for both the test and control lines was analyzed by the SIA. The exposure of the light was designed to be constant using the smartphone flash software algorithm. Images were captured only when the shooting focus was within the proper measuring distance, which was provided on the screen as the guidelines. All images were converted to color spectrum data by each color, and the pixel intensities of test line were calculated percentiles compared with control line intensities by same the color spectrum data. The mean pixel intensity of the test and control lines was determined and used to calculate the percent test to control pixel intensity ratio (T/C ratio) as follows: T/C ratio (%) = (test line pixel intensity/control line pixel intensity) × 100%. 

### 2.5. Statistical Analysis

The T/C ratios of the BZ-nAb were analyzed using receiver operating characteristic (ROC) analysis to select the optimal cutoff consistent with the visual interpretation results. The 95% confidence interval for sensitivity and specificity of the BZ-nAb and the cPass sVNT using PRNT-90 as the reference, were compared to evaluate their performance for the detection of SARS-CoV-2 nAbs. Agreement of the BZ-nAb and cPass sVNT results were verified using Cohen’s kappa coefficient (K), wherein the strength of agreement was defined as follows: K < 0, poor; 0–0.2, slight; 0.21–0.4, fair; 0.41–0.6, moderate; 0.61–0.8, substantial; and 0.81–1, almost perfect. The correlations between the values obtained from aforementioned three methods were analyzed using Pearson’s correlation analysis. Differences with *p*-values <0.05 were considered statistically significant. Statistical analyses were performed using MedCalc version 18.11.6 (MedCalc Software bvba, Ostend, Belgium), and visualizations were performed using MedCalc and R software (version 3.4.3).

## 3. Results

**Figure 2:**
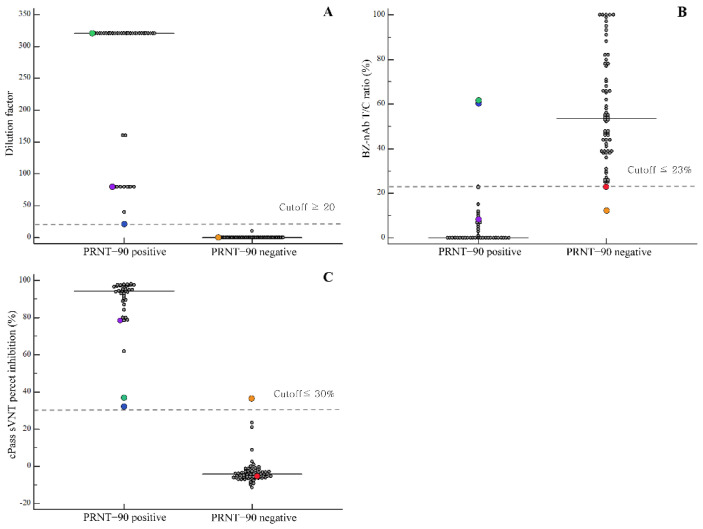
Data distribution and comparison between PRNT-90-positive and -negative groups. (**A**) PRNT-90 dilution factor; (**B**) BZ-nAb T/C ratio; (**C**) cPass sVNT percent inhibition. When compared with the PRNT-90 positive samples, the BZ-nAb showed three discordant results when visually interpreted (green, blue, purple dots), and two when interpreted using T/C ratio (green, blue dots). When compared with the PRNT-90 negative samples, the BZ-nAb showed two discordant results using T/C ratio (red, orange dots), and one discordant result was also noted with cPass sVNT (orange dot). The horizontal dashed lines indicate the cutoffs for each assay.

A total of 104 samples (45 COVID-19-positive samples and 59 COVID-19-negative samples) were used in this study. PRNT-90 was used as the reference for the detection of SARS-CoV-2 nAbs, and the samples were grouped based on the obtained results. Of the 45 COVID-19 samples, six samples showed negative PRNT-90 results and were, thus, included in the PRNT-90-negative group. Of the six samples with negative PRNT-90 results, five samples were collected less than 5 days after the symptom onset (2 samples on day 0, 1 sample on day 2, and 2 samples on day 4), and one sample was collected 10 days after the symptom onset. Of the 59 presumed COVID-19 negative samples, one sample showed a positive PRNT-90 result (1:40) and was included in the PRNT-90-positive group. In total, 40 and 64 samples were included in the PRNT-90-positive and -negative groups, respectively. The distribution of dilution factors for the PRNT-90-positive and -negative groups is shown in Figure 2A.

### 3.1. BZ-nAb Test Line Intensity Measurement by the SIA

**Figure 3:**
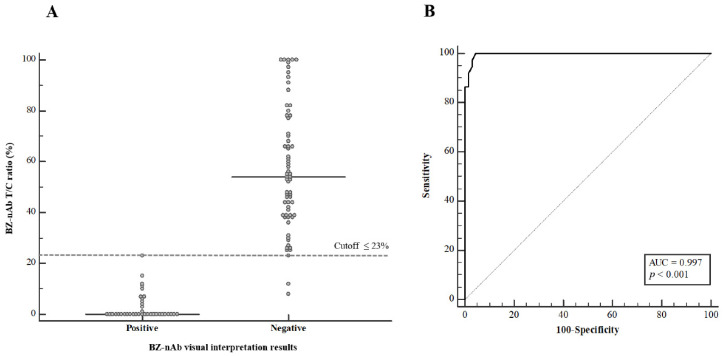
Comparison of the T/C ratio-interpreted and visually interpreted results of the BZ-nAb test. (**A**) Data distribution of BZ-nAb visually interpreted results. The horizontal dashed line indicates the cutoff selected using the receiver operating characteristic (ROC) analysis. (**B**) The ROC curve for the T/C ratio results, compared to the visually interpreted results. The area under the curve (AUC) values are also presented.

For all samples subjected to the BZ-nAb, the intensities of the test and control lines were measured using an SIA, and the T/C ratio was calculated. The median T/C ratio for the visually interpreted BZ-nAb positive and negative groups were 0% and 54.0%, respectively (Figure 3A). The 90th percentile T/C ratio for the BZ-nAb visually interpreted positive and negative groups was 13.5% and 97.2%, respectively. ROC analysis of the T/C ratio showed that a cutoff equal to or lower than 23% were visually interpreted positive BZ-nAb samples with a sensitivity of 100% and a specificity of 95.5% (area under curve 0.997) (Figure 3B).

### 3.2. Performance Evaluation of the BZ-nAb and cPass sVNT for the Detection of SARS-CoV-2 nAbs

The estimated diagnostic performances of BZ-nAb and cPass sVNT are shown in Table 1. For the BZ-nAb, the effect of the T/C ratio cutoff obtained by ROC analysis (cutoff ≤ 23%) was also analyzed. The BZ-nAb showed a sensitivity ranging from 92.5% to 95.0% and a specificity of 96.9% to 100%. Interpretation using the T/C ratio increased BZ-nAb sensitivity but lowered specificity. The cPass sVNT was the more sensitive method compared to the BZ-nAb, regardless of the interpretation method. However, the specificity of the cPass sVNT was lower than that of the BZ-nAb when results were visually interpreted (Table 1).

Comparisons between PRNT-90-positive and -negative groups with respect to the distributions of dilution factor for PRNT-90, T/C ratio for the BZ-nAb, and percent inhibition for the cPass sVNT are shown in Figure 2. For PRNT-90-positive and -negative groups, the median T/C ratios were 0% and 53.5%, and the median percentage inhibition was 94.2% and −4.2%, respectively. Among the PRNT-90-positive samples, three samples were false-negatives according to the results of visual interpretation of BZ-nAb (Figure 2 and Figure S2A, green, blue, and purple dots), and two samples were also false-negatives when interpreted using the T/C ratio cutoff (Figure 2 and Figure S2A, blue and green dots). These two false-negative samples showed PRNT-90 titers of 1:20 and above 1:320 and were positive for cPass sVNT with relatively low cPass sVNT percentage inhibition values (31.2% and 36.1%, respectively). One PRNT-90 negative sample was a false-positive according to both the BZ-nAb using T/C ratio cutoff and the cPass sVNT; however, this sample was negative according the BZ-nAb visually interpreted result (Figure 2 and Figure S2B, orange dot). The dilution factor of PRNT-90 was more strongly correlated with cPass sVNT percent inhibition than with the T/C ratio of the BZ-nAb (r = 0.8660 vs. r = −0.7089; both *p* < 0.001). 

### 3.3. Agreement of BZ-nAb and cPass sVNT Results

**Figure 4:**
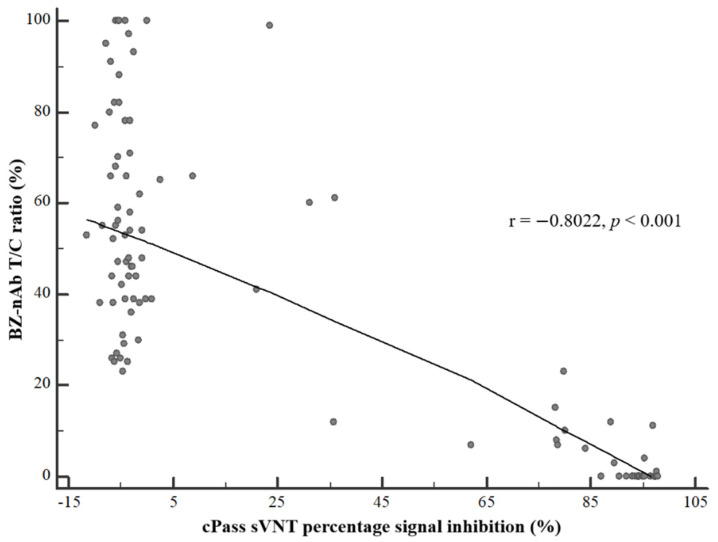
Correlation between the BZ-nAb T/C ratio and cPass sVNT percent inhibition. Pearson’s correlation analysis showed a strong negative correlation between BZ-nAb T/C ratio and cPass sVNT percent inhibition (r = −0.8022, *p* < 0.001) when analyzed using a total of 101 samples.

An almost perfect agreement was observed between the results of cPass sVNT and BZ-nAb with visual interpretation (κ = 0.914, 95% confidence interval (CI): 0.831–0.996) and that of the cPass sVNT and BZ-nAb with T/C ratio interpretation (κ = 0.936, 95% CI: 0.866–1). The results of the BZ-nAb with T/C ratio interpretation showed a higher positive agreement, but lower negative agreement, with cPass sVNT results compared to the results of the BZ-nAb test obtained by visual interpretation (Table 2). There was a strong negative correlation between BZ-nAb T/C ratio and cPass sVNT percent inhibition (r = −0.8022, *p* < 0.001) (Figure 4).

## 4. Discussion

Rapid and accurate SARS-CoV-2-specific nAb measurement can provide additional insight into understanding the development of host immunity against COVID-19 and vaccine development and aid in characterizing convalescent plasma for therapy. To the best of our knowledge, the BZ-nAb is the first RDT designed to detect SARS-CoV-2 nAbs based on the neutralizing capacity of nAbs. RDTs can be rapidly and easily performed, contributing towards its feasibility as standard SARS-CoV-2 nAb detection tests; however, they show varying degrees performance. In this study, we evaluated the diagnostic performance of the BZ-nAb by comparing it to that of the cPass sVNT, using the PRNT-90 as a reference. In terms of SARS-CoV-2 nAb detection, the BZ-nAb showed a diagnostic performance comparable to that of the cPass sVNT, although the sensitivity of the BZ-nAb was slightly lower than that of the cPass sVNT. 

The overall performance of the BZ-nAb was acceptable, regardless of the interpretation method. Although the correlation between dilution factor of PRNT-90 and T/C ratio of BZ-nAb was strong, the impact of dilution factors on the false negative samples was not definite. The two false negative samples, which were negative both as a result of visual and T/C ratio interpretation but positive according to the PRNT-90, showed a wide range of dilution factors (20 and over 320). Interestingly, the discordant sample with a PRNT-90 titer of > 1:320 also showed a relatively low cPass sVNT percentage inhibition value, indicating the possible presence of non-RBD targeting SARS-CoV-2 nAbs in this sample. SARS-CoV-2 nAbs that target proteins other than RBD, such as the N-terminal domain of the S protein, have been isolated in COVID-19 patients [22]. 

The performance of the cPass sVNT showed high sensitivity and specificity when the PRNT-90 was used as the reference standard. These results are consistent with a previous study that had also performed an evaluation based on the PRNT-90 results [15]. Papenburg et al. also reported perfect sensitivity of the cPass sVNT but various specificities depending on the reference standard used. When compared with the PRNT-50 results, cPass sVNT showed specificities ranging from 91% to 100%, but, when compared with PRNT-90 results, the specificities ranged from 57% to 61%. The authors postulated that protocols for the same reference methods could potentially vary across laboratories [23]. The performance of cPass sVNT, especially its specificity, requires further investigation. 

Visual interpretation of RDTs is subjective with variable results depending on the interpreter. Analyzing RDTs with SIA has been reported to provide improved and reliable results [18,19,20]. In this study, when the signal intensity of BZ-nAb was measured using the SIA, the T/C ratio cutoff led to an increase in the sensitivity of the BZ-nAb from 92.5% to 95.0%, impacting its performance, although at the expense of its specificity. Another advantage of quantifying signal intensity using the T/C ratio was that the correlation between the PRNT-90 dilution factor and cPass sVNT percent inhibition value could be analyzed. The BZ-nAb showed a stronger correlation with the cPass sVNT compared to that with the PRNT-90, possibly because of the similarities between the two assays, such as use of purified RBD of S protein and detection of the inhibition of the RBD-ACE2 interaction by nAbs. 

This study has limitations. First, a relatively small number of COVID-19 patient sera was evaluated, and longitudinal follow-up could not be carried out due to the lack of availability of samples at different time points. Secondly, the cross-reactivity of BZ-nAb evaluation using the sera containing antibodies against other coronaviruses or syphilis was not performed. Furthermore, while we used the PRNT-90 as the reference, PRNT with other percentages of plaque reduction, such as PRNT-50, were not available for further evaluation. The PRNT is considered the standard method for SARS-CoV-2 nAb detection by many regulatory agencies, but the guidelines do not specify the percentage of plaque reduction that should be evaluated. The more stringent PRNT-90 value selected in the current study for evaluating the diagnostic performances using the COVID-19 patient sera, could had impact in high sensitivity observed in our study. Our results should be interpreted considering that the PRNT-90 has been used as the reference and could potentially impact the performance. Future research assessing both PRNT-50 and PRNT-90 will be important to examine the BZ-nAb performance for samples with discrepant results between PRNT-50 and PRNT-90 discrepant results, especially PRNT-50 positive and PRNT-90 negative samples. 

## 5. Conclusions

Our performance evaluation of the BZ-nAb demonstrated its ability to detect SARS-CoV-2 nAbs in COVID-19 patients. The BZ-nAb was found to have a performance comparable to that of the cPass sVNT. An almost perfect agreement was observed between the cPass sVNT and BZ-nAb results. SIA was useful because the application provided improved sensitivity and quantified signal intensity. Measurement of signal intensity of BZ-nAb using the T/C ratio was associated with an increased sensitivity of BZ-nAb and showed a strong correlation with the PRNT-90 dilution factor and a very strong correlation with cPass sVNT results. 

## Figures and Tables

**Table 1:** Performance comparison of the BZ COVID-19 neutralizing antibody test and cPass surrogate viral neutralization assay.

Neutralizing Ab Detection Methods	Total Number of Samples		True Positive	False Positive	False Negative	True Negative	Sensitivity ^a^ (95% CI)	Specificity ^b^ (95% CI)
	PRNT-90 Positive	PRNT-90 Negative						
BZ COVID-19 neutralizing Ab test								
Visual interpretation	40	64	37	0	3	64	92.5 (79.6–98.4)	100 (94.4–100)
Interpretation using T/C ratio cutoff	40	64	38	2	2	62	95.0 (83.1–99.4)	96.9 (89.2–99.6)
cPass surrogate virus neutralization test	37	64	37	1	0	63	100 (90.5–100)	98.4 (91.6–100)

Ab, antibody; CI, confidence interval; PRNT-90, 90% plaque reduction neutralization test; T/C ratio, test to control pixel intensity ratio. ^a^ (True positive/(True positive + False negative)) × 100 using PRNT-90 as the reference. ^b^ (True negative/(True negative + False positive)) × 100 using PRNT-90 as the reference.

**Table 2:** Agreement between BZ COVID-19 neutralizing antibody test and cPass surrogate viral neutralization assay.

		cPass sVNT Results		Percent Agreement (95% CI) ^a^			Kappa Statistic (95% CI)
		Positive	Negative	Positive	Negative	Overall	
BZ-nAb with visual interpretation	Positive	34	0	89.5 (75.2–97.1)	100 (94.3–100)	96.0 (90.2–98.9)	0.914 (0.831–0.996)
	Negative	4	63				
BZ-nAb with T/C ratio cutoff	Positive	36	1	94.7 (82.3–99.4)	98.4 (91.5–100)	97.0 (91.6–99.4)	0.936 (0.866–1)
	Negative	2	62				

BZ-nAb, BZ COVID-19 neutralizing antibody test; CI, confidence interval; cPass sVNT, cPass surrogate viral neutralization assay; T/C ratio, test to control the pixel intensity ratio. ^a^ Positive agreement was calculated (BZ-nAb and cPass sVNT positive/cPass sVNT positive) × 100, negative agreement was calculated (BZ-nAb and cPass sVNT negative/cPass sVNT negative) × 100, and overall agreement was calculated (positive or negative by both BZ-nAb and cPass sVNT/(cPass sVNT positive + cPass sVNT negative)) × 100.

## Data Availability

Not applicable.

## Supplementary Materials

The following are available online at https://www.mdpi.com/article/10.3390/diagnostics11122193/s1, Figure S1: BZ-nAb and the T/C ratio measurement using a smartphone-based image analysis application (SIA). Figure S2: Data distribution of PRNT-90 dilution factor, BZ-nAb T/C ratio, and cPass sVNT percent inhibition in PRNT-90-positive and -negative groups.
