# Supplementary material for: Performance Evaluation of the BZ COVID-19 Neutralizing Antibody Test for the Culture-Free and Rapid Detection of SARS-CoV-2 Neutralizing Antibodies
Source: Diagnostics (Basel). 2021 Nov 25;11(12):2193. doi: 10.3390/diagnostics11122193 (PMC8700253; doi:10.3390/diagnostics11122193)
Supplement: Supplementary file 1 [file diagnostics-11-02193-s001.zip › diagnostics-1458861-supplementary.pdf]

diagnostics

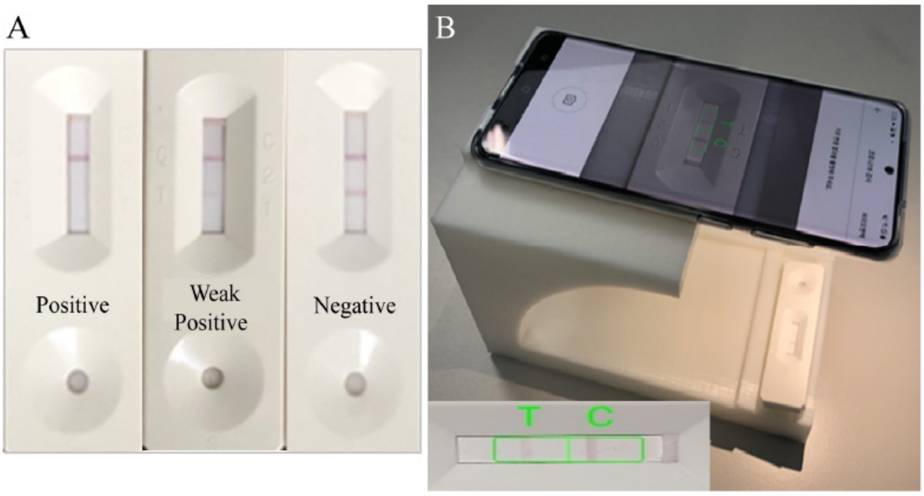

Figure S1. BZ-nAb and the T/C ratio measurement using a smartphone-based image analysis application (SIA). (**A**) Positive, weak-positive, and negative results observed in the BZ-nAb. (**B**) The image representing the BZ-nAb result was analyzed using the SIA by placing the BZ-nAb test line within the "T" green box and the BZ-nAb control line within the "C" green box. Then, the pixel intensities of the lines were analyzed using the SIA**.**

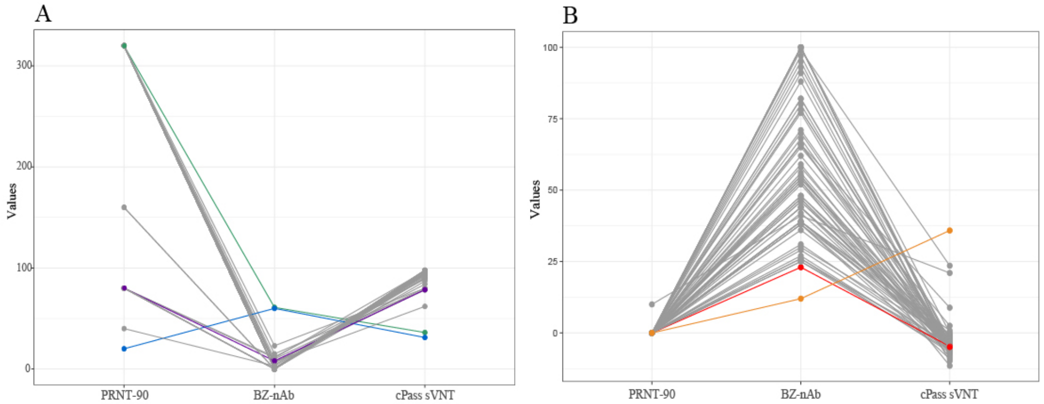

Figure S2. Data distribution of PRNT-90 dilution factor, BZ-nAb T/C ratio, and cPass sVNT percent inhibition in PRNT-90-positive (**A**) and -negative (**B**) groups. In PRNT-90 positive samples, the BZ-nAb showed three discordant results when visually interpreted (green, blue, purple dots), and two when interpreted using T/C ratio (green, blue dots). In the PRNT-90 negative samples, the BZ-nAb showed two discordant results using T/C ratio (red, orange dots), and one discordant result was also noted with cPass sVNT (orange dot).
